# *Thorsellia* dominance and bacterial community structure in two major malaria vectors: an exploratory study in Northwestern Argentina

**DOI:** 10.3389/fmicb.2026.1895126

**Published:** 2026-09-23

**Authors:** Paul Leonardo Duque, María Victoria Micieli, Ximena Pérez-Otáñez, Gabriela María Silenzi-Usandivaras, Cristian Laci, María Julia Dantur-Juri

**Affiliations:** 1 Unidad Ejecutora Lillo (CONICET-Fundación Miguel Lillo), San Miguel de Tucumán, Tucumán, Argentina; 2 Centro de Estudios Parasitológicos y de Vectores (CEPAVE-CCT-La Plata- CONICET-UNLP), La Plata, Argentina; 3 Facultad de Medicina Veterinaria y Agronomía, Universidad UTE, Quito, Ecuador; 4 Grupo de Investigación CABSAC, Facultad de Medicina Veterinaria y Agronomía, Universidad UTE, Quito, Ecuador; 5 Instituto de Genética y Microbiología, Fundación Miguel Lillo, San Miguel de Tucumán, Tucumán, Argentina; 6 Coordinación Nacional de Control de Vectores, Ministerio de Salud de la Nación, Salta, Argentina

**Keywords:** *An. pseudopunctipennis*, *Anopheles argyritarsis*, Argentina, microbiota, mosquito

## Abstract

Malaria continues to be one of the major global health burdens despite worldwide efforts to reduce its incidence and prevalence. Recent technological advances have generated new mosquito-control alternatives, one of which involves the use of symbionts that are part of the core gut microbiota to develop paratransgenesis strategies. This exploratory study characterized the bacterial community of *Anopheles argyritarsis* and *Anopheles pseudopunctipennis* across three environments: Iruya River (mountain forest), Conchas River (transitional zone), and Chirimayo River (agricultural landscape). Through 16S rRNA gene sequencing, we obtained 1,308 amplicon sequence variants (ASVs). We registered dominance of the phyla Proteobacteria (94%) and Bacteroidota (3.28%). At the genus level, *Thorsellia* was the dominant taxon within the core microbiota (71.5%). Shannon (5.09–5.95) and Simpson (0.988–0.996) diversity indices indicated that, despite the increase of multiple bacterial genera in anthropized areas, community evenness remained balanced. Meanwhile, beta diversity analysis (Bray-Curtis) demonstrated that both host species and sampling area influenced bacterial composition. These results are presented within a descriptive and exploratory framework due to the limited replication across species-locality combinations. Despite the challenge of establishing host-symbiont relationships, *Thorsellia* is a genus that warrants further tracking across different developmental stages based on its notable stability in this study. Under the holobiont perspective, understanding how the microbiota influences vector fitness, longevity, and resilience will help guide different efforts toward control and surveillance strategies across ecological settings for future investigations. Although Argentina has not reported autochthonous malaria cases, the vector persists and the risk of re-emergence remains latent.

## 1 Introduction

Malaria continues to be one of the major global health burdens. In the region of the Americas, in 2024, there were 663,000 recorded cases and 504 deaths (World Health Organization, 2025). In Argentina, since the certification in 2019 as free of autochthonous transmission, no local transmission has been recorded to date (World Health Organization, 2019, 2025). Nevertheless, the risk of reintroduction persists due to the proximity to endemic border countries, such as Bolivia, which experienced a surge and an increase in both incidence and mortality of over 70%, and Brazil, one of the three countries accounting for more than 75% of cases in the region (World Health Organization, 2025).

Historically, in northwestern Argentina, *Anopheles pseudopunctipennis* and *An. argyritarsis* were the primary and secondary vectors, respectively, involved in the transmission of *Plasmodium vivax* infections (Zaidenberg, 2015). This parasite is responsible for almost two-thirds of cases in the region, transmitted through the bite of infected female *Anopheles*.

Despite the different ways to control and reduce populations of mosquitoes, we still require new alternatives due to the emergence of insecticide-resistant mosquito strains (World Health Organization, 2021). Recent advances in Next-Generation Sequencing technology have helped us understand and use helpful microorganisms that naturally live inside (symbionts) the mosquito's midgut (Fofana et al., 2021; Gendrin and Christophides, 2013; Nanfack Minkeu and Vernick, 2018). Some researchers have been using the symbionts with good results in laboratory and semi-field conditions (Fofana et al., 2022; Kotnis and Kuri, 2016; Mancini et al., 2016). Among symbiont-based control strategies in anophelines, the use of extracellular bacteria from the genus *Asaia* stands out. Its stability and capacity for transstadial transmission consolidate it as an ideal candidate for paratransgenesis strategies (genetic modification of the symbiont to block parasite development in the adult) (Favia et al., 2008; Capone et al., 2013).

Despite reports of transstadial transmission of the bacterial genus *Thorsellia* (isolated for the first time from the gut of *An. arabiensis*) (Kampfer et al., 2006), its consolidation as a symbiont for control strategies remains under debate and underscores the complex host-symbiont interactions. Apparently, this bacterium shares a close symbiotic and evolutionary relationship with *Anopheles* (Briones et al., 2008). Although it has been reported as a frequent and/or dominant genus in the core microbiota of many *Anopheles* species, particularly in larval stages and recently emerged adults (Briones et al., 2008; Wang et al., 2011; Gendrin and Christophides, 2013; Duque-Granda et al., 2025), other studies have highlighted the challenges of selecting this symbiont due to metamorphosis, during which a large portion of the bacterial communities is reduced or replaced (Romoser et al., 2000; Moll et al., 2001), maintaining the hypothesis that it may be an opportunistic colonizer adapted to the niche (Rani et al., 2009). Another aspect highlighting its complexity is strain heterogeneity (not all strains are capable of surviving metamorphosis) and limited genomic information (Briones et al., 2008).

In recent decades, biotechnological advances have diversified fields of exploration, including the characterization of microbiota with a focus on vector control strategies. However, this approach in anophelines in Argentina presents an information gap, being limited to targeted PCR detection, culture-dependent methods, or studies in other mosquito genera. To our knowledge, this study represents the first characterization through massive sequencing of 16S gene amplicons of the gut microbiota of *An. pseudopunctipennis* and *An. argyritarsis* in Argentina and the Americas, as well as the first study in Argentina focused on species that naturally cohabit across different levels of human intervention in their surrounding landscapes. The research provides, in an exploratory manner, a baseline for bacterial community ecology in the immature stages of two medically important mosquitoes within areas with a historical record of malaria transmission in a post-certification country, laying the groundwork for future research on integrated vector management.

## 2 Material and methods

### 2.1 Study sites and mosquito processing

The study was conducted in three areas of Northwestern Argentina (Figures 1A, B), during the warm, wet season spanning the end of 2023 and the beginning of 2024, a period characterized by elevated, sporadic rainfall events in the region. The sampling covers a significant latitudinal gradient of ecological areas with a history of malaria transmission (Dantur-Juri et al., 2009; Carter, 2012; Arana et al., 2021).

**Figure 1:**
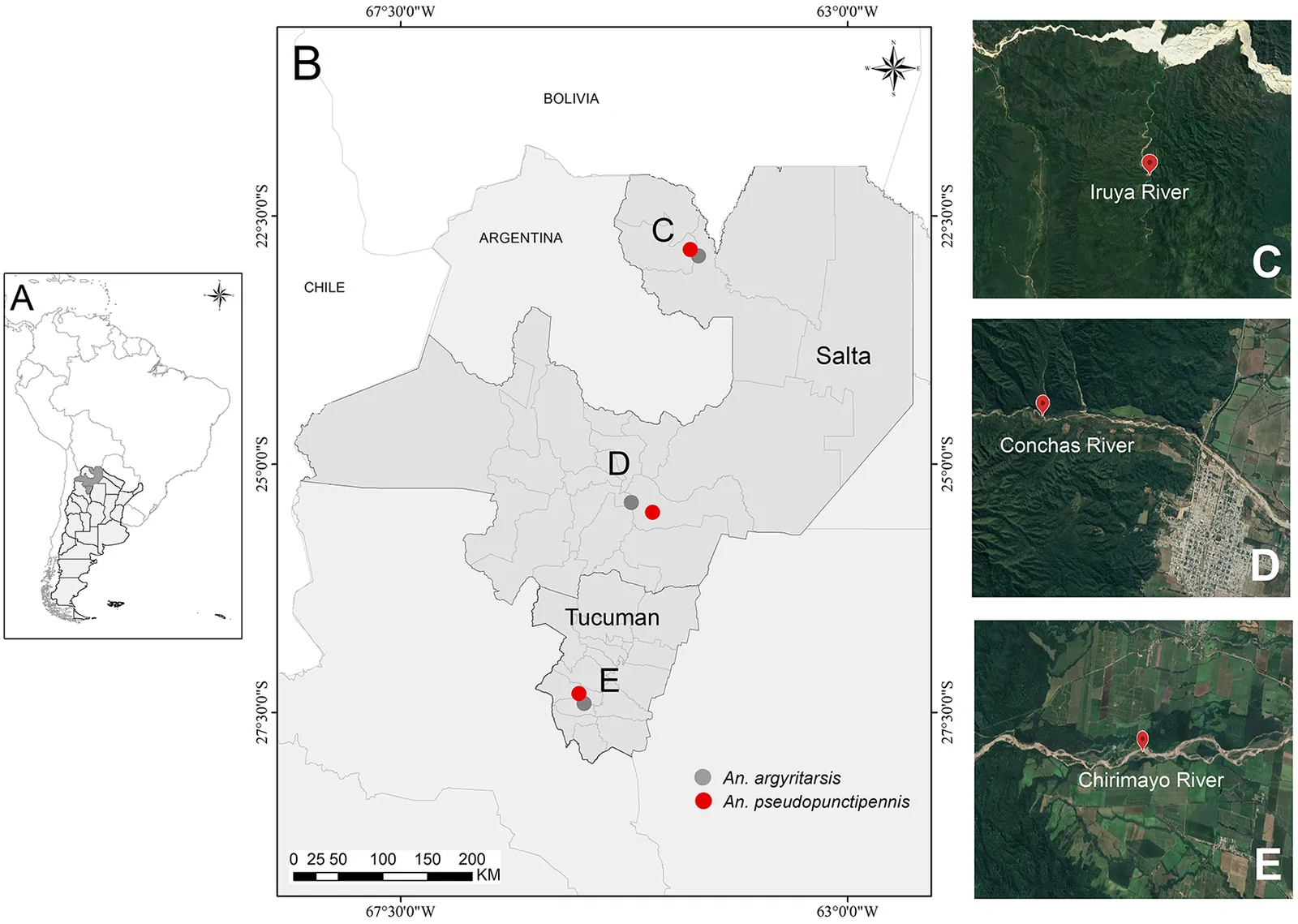
Study sites in Northwestern Argentina. **(A)** Map showing the location of Argentina (in greyscale) within South America. **(B)** Geographic distribution of *An. argyritarsis* and *An. pseudopunctipennis* collection sites in Salta and Tucuman provinces. **(C)** Iruya River area, characterized by dense vegetation in a mountain terrain. **(D)** The Conchas River area, located near Metán town. **(E)** Chirimayo River area, showing a fragmented landscape due to human activity.

The Iruya River (Salta province) is located in a mountainous piedmont forest, surrounded by dense vegetation cover (Figure 1C). The watercourse is characterized by a broad channel and a constant flow. Breeding sites were located along the river margins, where the sinuosity of the banks favors the formation of pools among vegetation. These habitats presented partial solar exposure, clear and non-turbid water, and a high abundance of *Spirogyra* sp. algae and aquatic and terrestrial vegetation, resting over a predominantly sandy substrate. The Conchas River (Salta province) is an ecological transition zone with dense vegetation, near the town of Metán. Its landscape is fragmented by bridges, pathways, and small farms (Figure 1D). The watercourse was characterized by a slow flow, with stagnant water accumulating along the banks. The habitat was fully exposed to sunlight and featured clear, non-turbid water, with algae and floating vegetation over a substrate composed of clay, sand, and large rocks. Finally, the Chirimayo River (Tucumán) is located in a fragmented forest with high agricultural influence (Figure 1E). Its watercourse was slow and constant. The breeding sites were characterized by the presence of large submerged rocks, among terrestrial and aquatic vegetation. The habitat was exposed to sunlight, with a sandy and rocky substrate.

To characterize the basic conditions of the habitat, water temperature was measured at each breeding site using a digital thermo-hygrometer (Springfield Precision Instruments Inc., Wood Ridge, NJ), recording 24 °C in the Iruya River, 21 °C in the Conchas River, and 22 °C in the Chirimayo River. Water pH was determined using pH indicator strips (MQuant^®^, Merck, Darmstadt, Germany) and ranged between 7 and 8 in all samples.

Larvae were collected using the standard dipping technique (300 ml) along the vegetation and ponds of each river (World Health Organization, 1975; Service, 1993). Collected larvae were preserved in sterile containers with water from the habitat, transported to the laboratory, and identified using taxonomic keys (Forattini, 2002; Sallum et al., 2020), while maintaining sterile conditions (Fierer et al., 2025). Of the identified material, a total of 38 individuals were stored in six pools (three per species, 6–7 individuals each) in sterile 1.5 mL vials containing 70% ethanol at −20 °C until processing. The pooling strategy was selected based on the larval co-habitation patterns and abundance across sampling sites, serving as the optimal approach given the logistical and ecological constraints inherent to field collection across these remote sites.

### 2.2 Tissues collection and DNA extraction

The guts of 38 larvae were dissected under sterile conditions. The larvae were placed in 96% ethanol for 3 min, then washed twice in sterile saline solution. Using sterile forceps, the guts were extracted and stored in pooled groups of six to seven in sterile vials containing 70% ethanol until processing in the laboratory (Galeano-Castañeda et al., 2019; Fierer et al., 2025). Genomic DNA was extracted using the DNeasy Blood & Tissue kit (Qiagen, Hilden, Germany) following the manufacture's recommendations. The extracted gDNA was quantified (NanoDrop, NanoDrop Technologies, Montchanin, DE, USA) and preserved at −20 °C.

### 2.3 16S rRNA gene amplification and sequencing

PCR amplification was performed targeting the V3–V4 hypervariable regions of the bacterial 16S rDNA gene using the primers 341F (CCTACGGGNGGCWGCAG) and 805R (GACTACHVGGGTATCTAATCC). PCR and library preparation were carried out following the Macrogen 16S Metagenomic Sequencing Library preparation protocol. Sequencing was performed by Macrogen Inc. (Seoul, South Korea) through the Illumina MiSeq platform with a 2 x 300 bp paired-end configuration (targeting ~460 bp amplicons).

### 2.4 Bioinformatic processing

Raw reads were processed using the DADA2 pipeline (Callahan et al., 2016) in R v4.5.2 software (R Core Team, 2021). The quality of the reads was filtered and trimmed with the parameters *truncLen* = *c*(280, 240), *maxN* = 0, *maxEE* = *c*(2, 2), *truncQ* = 2, and the activation of PhiX contamination removal. Error rates were estimated from the filtered reads, followed by dereplication. Amplicon Sequence Variants (ASVs) were inferred using pooled sample inference (pool = TRUE) (Reitmeier et al., 2021). Paired reads were merged, and an ASV table was constructed. We retained fragments between 440 and 466 bp. Finally, chimeric sequences were removed using the consensus method, thus obtaining the final ASV table. Taxonomy was assigned using the naïve Bayesian classifier against the SILVA v138 database.

Due to the absence of negative controls, the ASV matrix was depurated based on a minimum relative abundance threshold of 0.01% and a prevalence in at least two samples (Callahan et al., 2016; Davis et al., 2018; Reitmeier et al., 2021). These filters follow recommendations for filtering low abundance and prevalence to reduce stochastic noise and spurious sequences (Bokulich et al., 2013; Reitmeier et al., 2021). Additionally, rare taxa representing transient environmental bacteria were removed (Salter et al., 2014; Glassing et al., 2016; Eisenhofer et al., 2019). This filtering did not distort the alpha diversity patterns (comparing the original and filtered ASV matrices, data not shown), suggesting the removal of low-abundance noise rather than biologically meaningful taxa.

### 2.5 Statistical analysis

Raw sequence counts were rarefied to standardize sampling effort (we also conducted the analysis without rarefaction, with no chance in the basis of the results), to the minimum library size (15,991 reads). Rarefaction curves were generated to assess sequencing depth sufficiency. Four Alfa diversity metrics were evaluated: Observed richness, Chao 1, Shannon, and Simpson. Because only one species-locality combination was analyzed per group, a descriptive framework without inferential statistical testing is presented.

Beta diversity (1,308 ASVs) was calculated using Bray-Curtis index. To visualize compositional relationship, Principal Coordinates Analysis (PCoA), Non-metric Multidimensional Scaling (NMDS) ordination, a dendrogram, and heatmap approaches were used. The stress value was evaluated to assess goodness of fit and reliability of the dimensional representation. Community composition was explored through PERMANOVA (999 permutations) and multivariate dispersion (betadisper) to explore patterns in within-group variability. These analyses were exploratory rather than intended to test statistical hypotheses, due to limited replication within groups.

Finally, we explored different criteria of relative abundance thresholds and abundance to determine the core microbiota. We defined the core microbiota using a relative threshold of ≥0.1% (0.001) and a prevalence across all samples. Mean relative abundances of core genera were calculated across all samples, per mosquito species, and per sampling locality.

## 3 Results

A total of 181,661 reads were retained after DADA2 processing, grouped into 1,308 amplicon sequence variants (ASVs). Sequencing depth ranged from 15,991 to 45,577 reads per sample (Supplementary Table S1).

### 3.1 Composition of the bacterial community of the larval gut

The bacterial community was composed of 12 phyla, with Proteobacteria dominating at 94% of the total relative abundance. The remaining phyla showed lower abundances, including Bacteroidota (3.28%), followed by <1% for the rest.

At the species level, *Anopheles argyritarsis* was dominated by Proteobacteria (91.6%), followed by Bacteroidota (4.94%), and Firmicutes (1.5%), with the remaining phyla contributing <1%. Similarly, in *An. pseudopunctipennis*, the most representative was Proteobacteria (96.55%) and Bacteroidota (1.63%), followed by <1% in the remaining phyla. At the river level, the Conchas River showed a dominance of Proteobacteria (90.85%) and Bacteroidota (6.88%). In the Iruya River, the community reached the highest relative abundance of Proteobacteria (97.44 %), followed by <1% for the remaining phyla. In the Chirimayo River, Proteobacteria represented 93.94%, followed by Bacteroidota (2.29 %), and Firmicutes (2.06%), among others (<1%), all 12 phyla detected in the study were recorded here. Overall, slight differences in bacterial composition were observed among sampling areas (Figure 2, Supplementary Table S2).

**Figure 2:**
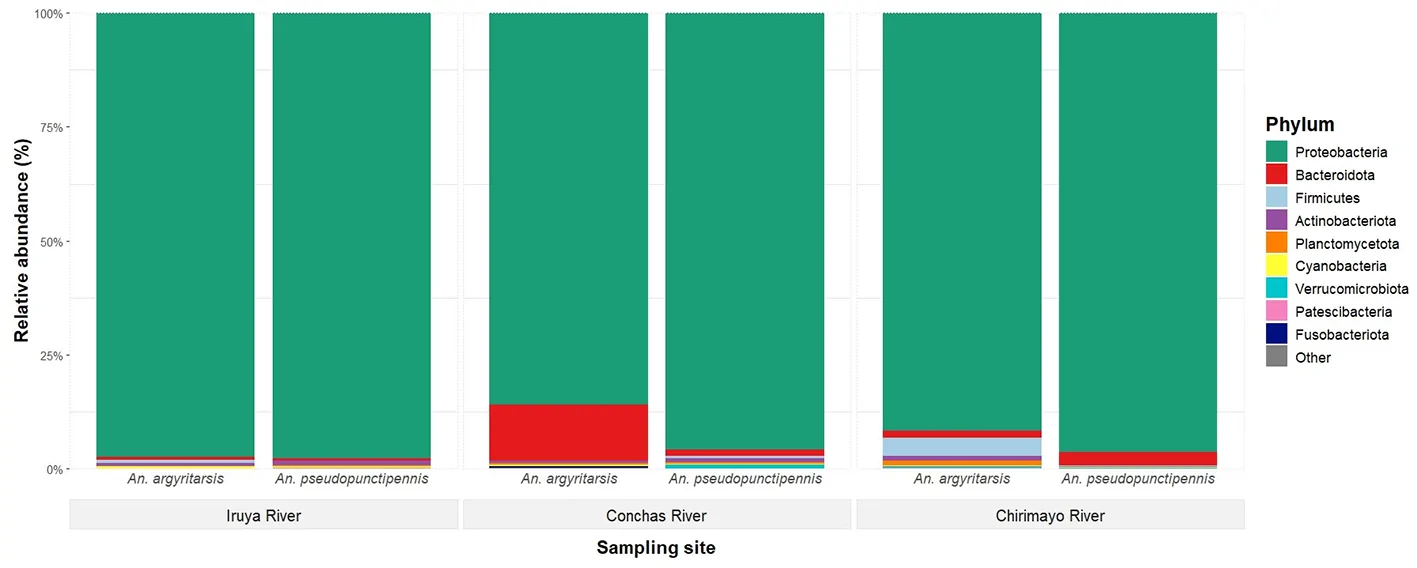
Bacterial community composition at the phylum level in *Anopheles argyritarsis* and *An. pseudopunctipennis* larvae. Stacked bars represent relative abundances based on ASVs, highlighting the prevalence of Proteobacteria in all three study areas (Northwestern Argentina).

The genus level was the basal taxonomic classification of this study. In *both An. argyritarsis* and *An. pseudopunctipennis*, the genus *Thorsellia* was the main colonizer, representing 71.5% across all samples. The remaining genera showed consistency, although at lower abundance. In *An. pseudopunctipennis, Thorsellia* was highly dominant (83.22%), while secondary genera appeared with abundances <1%. In contrast, *An. argyritarsis* showed a lower abundance of *Thorsellia* (59.83%), but a higher representation of other bacterial taxa (Table 1).

**Table 1:** Most abundant bacterial genera detected in mosquito larval guts, showing mean relative abundance across all samples (top 10) and the dominant taxa genera by species (top 5) and sampling area (top 5).

Phylum	Family	Genus	Mean abundance
Top 10 genera			
Proteobacteria	Thorselliaceae	*Thorsellia*	71.5
Proteobacteria	Comamonadaceae	*Ideonella*	3.45
Proteobacteria	Rhodobacteraceae	*Tabrizicola*	2.01
Proteobacteria	Comamonadaceae	*Hydrogenophaga*	1.80
Proteobacteria	Comamonadaceae	*Rhodoferax*	1.53
Proteobacteria	Rhodobacteraceae	*Gemmobacter*	1.50
Bacteroidota	Weeksellaceae	*Chryseobacterium*	1.24
Bacteroidota	Sphingobacteriaceae	*Nubsella*	1.17
Firmicutes	Exiguobacteraceae	*Exiguobacterium*	1.08
Proteobacteria	Dysgonomonadaceae	*Dysgonomonas*	0.54
Top 5–by species			
*Anopheles argyritarsis*			
Proteobacteria	Thorselliaceae	*Thorsellia*	59.83
Proteobacteria	Comamonadaceae	*Ideonella*	6.70
Proteobacteria	Comamonadaceae	*Hydrogenophaga*	2.84
Proteobacteria	Rhodobacteraceae	*Tabrizicola*	2.52
Proteobacteria	Comamonadaceae	*Rhodoferax*	2.43
*Anopheles pseudopunctipennis*			
Proteobacteria	Thorselliaceae	*Thorsellia*	83.22
Proteobacteria	Rhodobacteraceae	*Tabrizicola*	0.83
Proteobacteria	Dysgonomonadaceae	*Dysgonomonas*	0.76
Proteobacteria	Comamonadaceae	*Hydrogenophaga*	0.76
Proteobacteria	Rhodobacteraceae	*Gemmobacter*	0.65
Top 5–by river			
*Iruya river*			
Proteobacteria	Thorselliaceae	*Thorsellia*	90.54
Proteobacteria	Rhodobacteraceae	*Tabrizicola*	1.06
Proteobacteria	Comamonadaceae	*Hydrogenophaga*	0.37
Proteobacteria	Comamonadaceae	*Rhodoferax*	0.3
Proteobacteria	Rhodobacteraceae	*Gemmobacter*	0.3
*Conchas river*			
Proteobacteria	Thorselliaceae	*Thorsellia*	53.28
Proteobacteria	Comamonadaceae	*Ideonella*	6.96
Proteobacteria	Rhodobacteraceae	*Gemmobacter*	3.71
Bacteroidota	Weeksellaceae	*Chryseobacterium*	3.21
Bacteroidota	Sphingobacteriaceae	*Nubsella*	3.17
*Chirimayo river*			
Proteobacteria	Thorselliaceae	*Thorsellia*	70.83
Proteobacteria	Comamonadaceae	*Ideonella*	3.3
Proteobacteria	Comamonadaceae	*Hydrogenophaga*	3.09
Firmicutes	Exiguobacteraceae	*Exiguobacterium*	1.88
Proteobacteria	Comamonadaceae	*Rhodoferax*	1.18

Community composition varied among sampling areas. In the Iruya River, *Thorsellia* was by far the main colonizer (90.54%). In the Conchas River, the microbiota was more diverse with a greater representation of other taxa, *Thorsellia* dominated (53.28%), followed by *Ideonella, Gemmobacter, Chryseobacterium*, and *Nubsella*. Finally, in the Chirimayo River, the main colonizing genus was *Thorsellia* (70.83%) with substantial representation of *Ideonella, Hydrogenophaga, Exiguobacterium*, and *Rhodoferax* (Table 1, Figure 3).

**Figure 3:**
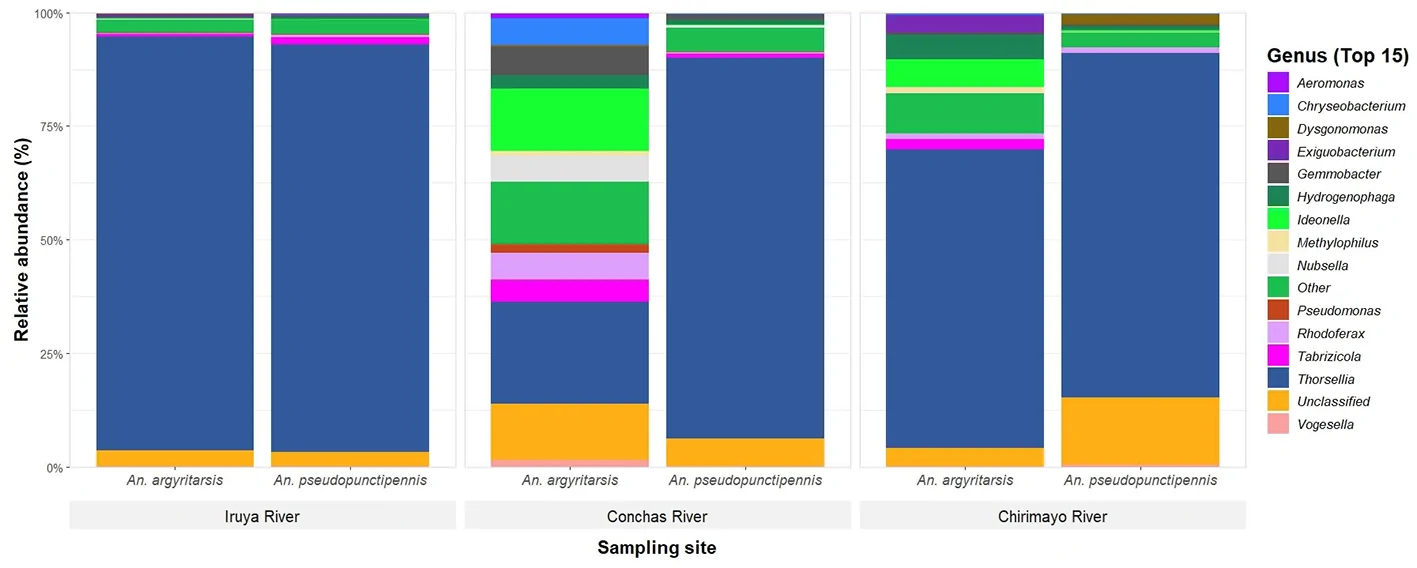
Genus-level taxonomic distribution of larval gut microbiota in *Anopheles argyritarsis* and *An. pseudopunctipennis*. Stacked bars represent the relative abundance of the 15 most frequent genera. *Thorsellia* (orange) is identified as the core dominant genus, followed by site-specific bacterial groups in the Iruya, Conchas, and Chirimayo rivers.

### 3.2 Diversity and microbial community structure of the larval gut

Sequences were rarefied to 15,991 reads per sample. Rarefaction curves showed a deceleration in ASV accumulation around 10,000 reads, suggesting this depth captured the majority of the diversity present in the six larval gut samples (Supplementary Figure S1).

Alpha diversity was evaluated through observed richness (number of ASVs), Chao1, and the Shannon and Simpson diversity indices. These metrics provide an overview of community structure by estimating the number of taxa present and the distribution of their relative abundances. Because only a single pooled sample was available for each species-locality combination, the results are descriptive and exploratory rather than statistically supported.

Observed richness ranged from 606 to 872 ASVs. The highest richness was recorded in *An. argyritarsis* from the Chirimayo River (the most anthropized area), whereas the lowest richness was observed in *An. argyritarsis* from Conchas River. Chao1 estimates showed higher values (610.1 to 1040.2), suggesting that the main structure of the community was captured, but some bacterial diversity remained uncaptured. Shannon (5.09–5.95) and Simpson (0.988–0.996) indices showed stable trends (Table 2), indicating that, despite the increase in the number of ASVs in anthropized areas, the evenness and overall structure of the microbial community remained relatively balanced. However, when taxonomic composition was examined, a strong dominance of the genus *Thorsellia* was evident. This suggested that although the communities contained multiple bacterial genera, the overall community structure was influenced by a number of taxa with similar abundances. Differences in alpha diversity among species and sampling rivers did not show drastic changes (Figure 4). The Conchas River community tended to exhibit higher Shannon values and lower relative dominance of *Thorsellia*, coinciding with a greater representation of secondary genera. In contrast, samples from the Iruya River were characterized by a dominance of *Thorsellia*, resulting in simpler community structures.

**Table 2:** Alpha diversity indices of gut bacterial communities across mosquito species and sampling localities.

Index	Iruya river		Conchas river		Chirimayo river	
	*An. argyritarsis*	*An pseudopunctipennis*	*An. argyritarsis*	*An. pseudopunctipennis*	*An. argyritarsis*	*An. pseudopunctipennis*
Observed	727	629	606	695	872	644
Shannon	5.15	5.17	5.97	5.17	5.35	5.05
Simpson	0.99	0.991	0.996	0.989	0.99	0.989

**Figure 4:**
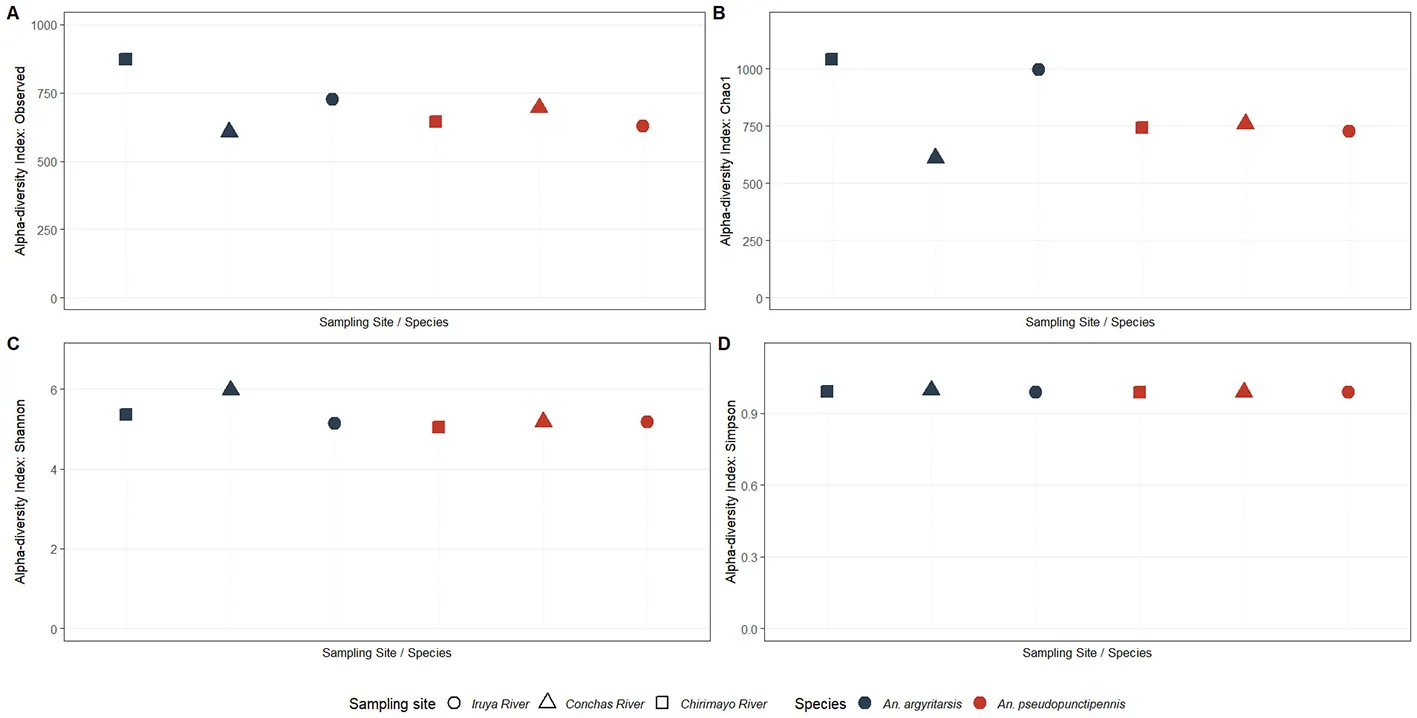
Alpha diversity indices of the larval gut microbiota of *Anopheles argyritarsis* and *An. pseudopunctipennis* across three river areas. Panels represent different diversity metrics: **(A)** Observed ASVs, **(B)** Chao1 richness estimator, **(C)** Shannon diversity index, and **(D)** Simpson dominance index. Symbols indicate the collection site (Square: Chirimayo River, Triangle: Conchas River, and Circle: Iruya River). Colors distinguish the mosquito species (*An. argyritarsis* in dark blue and *An. pseudopunctipennis* in red).

Bray-Curtis dissimilarity revealed structured variation, indicating that both area and species directly influence microbial composition. This pattern was evidenced in the PCoA ordination (Figure 5A), where the separation patterns showed heterogeneity among samples. The first two axes (45% and 34.8%) explained ~80% of the total variance. The most evident separation occurred between communities from the Iruya River and the Conchas River. NMDS analysis (Figure 5B) confirmed this with a stress value of 0.013, which indicated an excellent two-dimensional representation of the multivariate relationships. Both approaches consistently differentiate the bacterial communities, highlighting the natural forest area (Iruya River) and the area near the town (Conchas River). These findings were supported by the Bray-Curtis heatmap and dendrogram (Figure 6).

**Figure 5:**
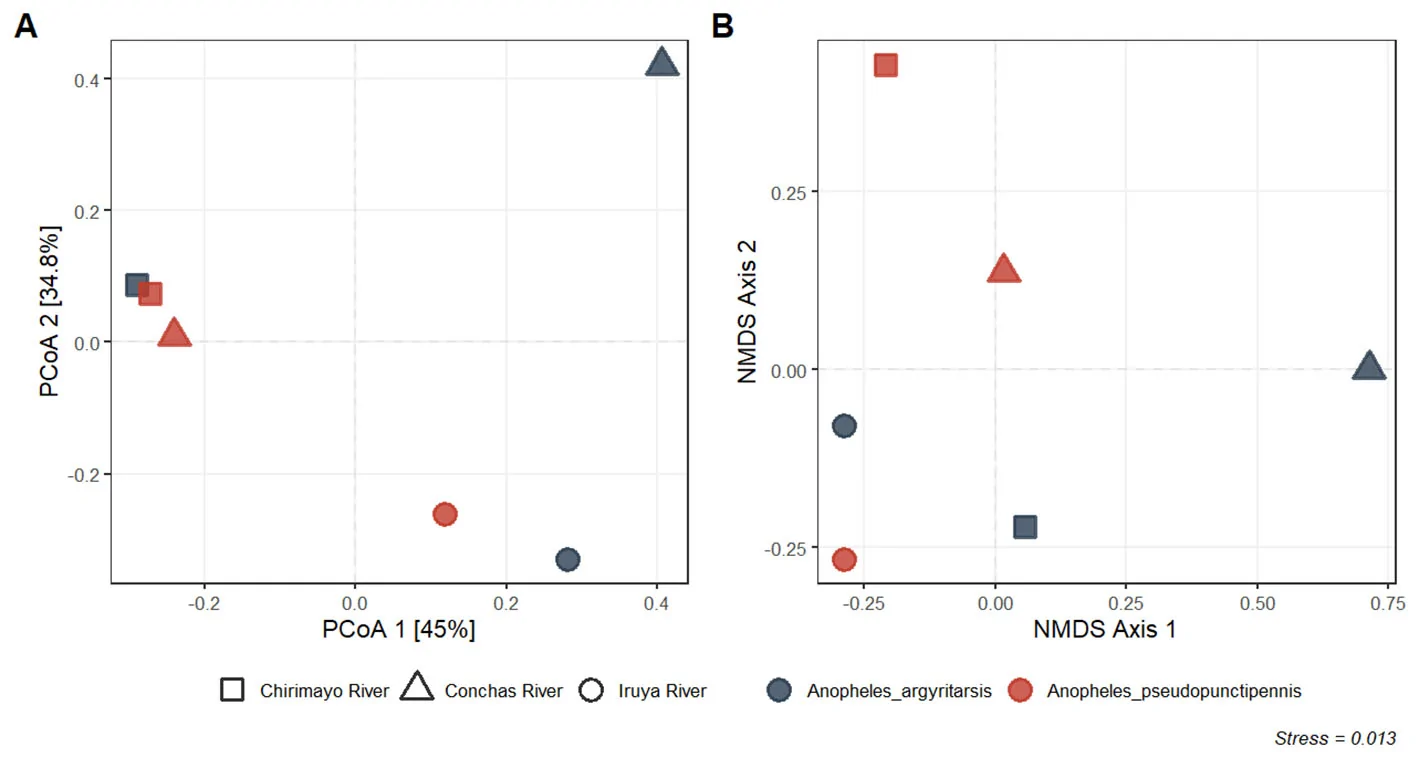
Community structure similarity (Beta diversity) of *Anopheles argyritarsis* and *An. pseudopunctipennis* larval microbiota. **(A)** PCoA and **(B)** NMDS plots based on Bray-Curtis dissimilarity. The low stress value in the NMDS (0.013) indicates an excellent representation of the data in two dimensions. Symbols represent river areas (Square: Chirimayo, Triangle: Conchas, and Circle: Iruya) and colors distinguish mosquito species.

**Figure 6:**
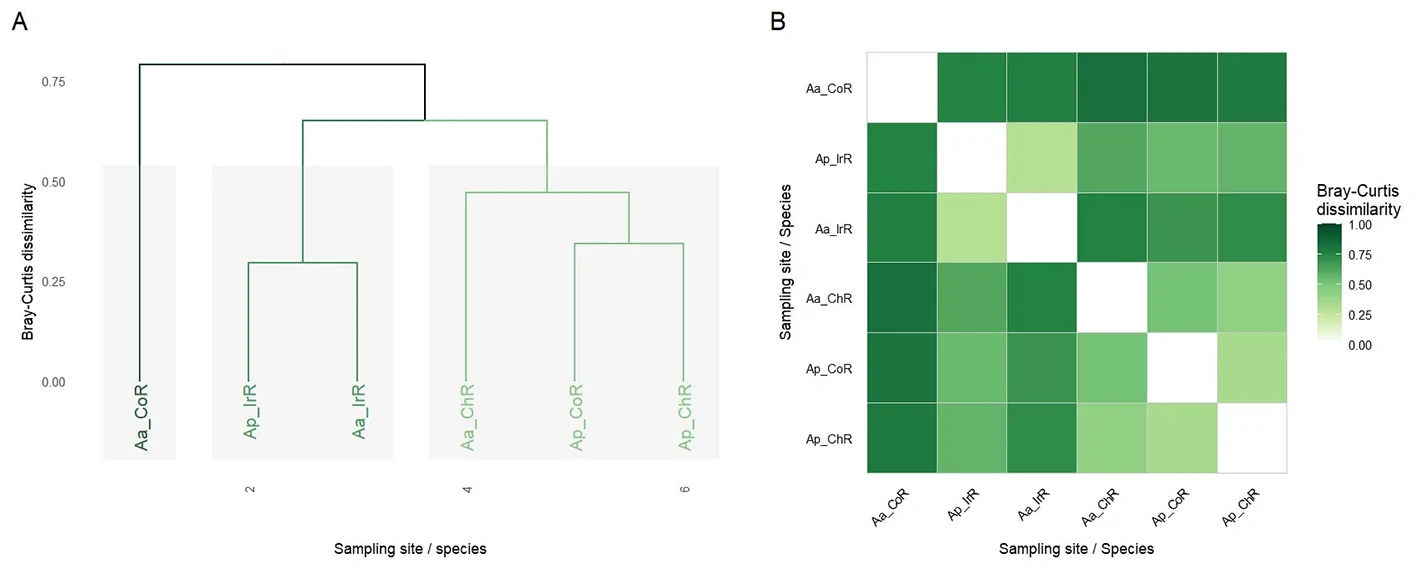
Hierarchical clustering and similarity analysis of larval gut bacterial communities based on Bray-Curtis dissimilarity. **(A)** Dendrogram illustrating the ecological distance between samples, where shorter branch lengths indicate higher community similarity. Gray shaded areas highlight the main clusters formed by river locality and species. **(B)** Heatmap displaying pairwise Bray-Curtis distances, with darker green shades representing higher dissimilarity (values closer to 0.8) and lighter shades indicating closer similarity. Aa, Anopheles argyritarsis; Ap, Anopheles pseudopunctipennis; IrR, Iruya River; ChR, Chirimayo River; CoR, Conchas River.

In this exploratory PERMANOVA, results indicated that both the host species and the sampling area were associated with differences in microbial communities, while beta-dispersion suggested no differentiation in within-group dispersion. Given the limited sample size and the lack of replicates, these results were indicative rather than confirmatory.

To establish a baseline for the composition of the core microbiota, a relative abundance threshold of >0.1% and a prevalence of 100% were applied. Under these criteria, five bacterial genera were identified as potential members of the strict and recurrent core microbiota (Supplementary Figure S2). Overall, the core community was strongly dominated by a single taxon, *Thorsellia*, which accounted for an average relative abundance of 71.5%. The remaining core genera were present at much lower abundances. In *An. argyritarsis* and *An. pseudopunctipennis* the core genera were dominated by *Thorsellia*, followed by *Hydrogenophaga, Rhodoferax, Gemmobacter*, and *Chryseobacterium*.

## 4 Discussion

This is the first exploratory study to describe the bacterial community in the larval gut of *An. pseudopunctipennis* and *An. argyritarsis* in Argentina. Our research focused on the larval phase as a critical developmental stage.

The analysis revealed the dominance of the phylum Proteobacteria, coinciding with investigations carried out on anophelines in Africa and the Neotropics (Wang et al., 2011; Gimonneau et al., 2014; Saab et al., 2020; Silva et al., 2021; Duque-Granda et al., 2025). In microbial studies of mountain lakes, the specific distribution of Proteobacteria was explained by local variations in dissolved organic matter and temperature (Newton et al., 2011; Gimonneau et al., 2014). Another key aspect is seasonality. In *Anopheles*, Proteobacteria dominated across both dry and wet seasons, reaching higher abundance in the wet season (Sandeu et al., 2022), whereas in other mosquito species, it dominated during the pre-monsoon season, with Firmicutes prevailing during the monsoon and post-monsoon seasons (Francis et al., 2024). The presence of Bacteroidota as the second most abundant phylum suggests that species feed on complex organic matter in constant degradation (Kirchman, 2006; Boissière et al., 2012). Although physicochemical water parameters were no evaluated, the detection of all 12 phyla in the Chirimayo River (agricultural landscape) suggests that disturbed environments may increase microbial diversity due to nutrient and organic matter enrichment. However, some authors maintain that genetic factors may be more determinant than environmental ones (Feng et al., 2021).

At the genus level, we found an extreme dominance of *Thorsellia* in both species, except for *An. argyritarsis* in the Conchas River. This aligns with a study in Colombia, where *Thorsellia* was identified as a member of core microbiome in *An. triannulatus s.l*. (Duque-Granda et al., 2025). Secondary genera (*Rhodoferax, Chryseobacterium*, and *Hydrogenophaga*) are part of the recurrent or most abundant core in various vectors (Coon et al., 2014; Muturi et al., 2017; Francis et al., 2024; Duque-Granda et al., 2025). Specifically, *Chryseobacterium* has been described as an essential symbiont for larval development and growth, as well as an inhabitant of soil, plant surfaces, and different depth layers within the same aquatic body (Coon et al., 2014; Gimonneau et al., 2014). The prevalence of *Thorsellia* in all processed samples is consistent, although its stability as a symbiont throughout the life cycle cannot yet be confirmed. This highlights the need for further longitudinal studies across different seasons and developmental stages to clarify whether this bacterial taxon represents a persistent symbiont or an opportunistic colonizer highly adapted to the gut niche.

At the locality level, the Iruya River showed an absolute dominance of *Thorsellia* (90%), suggesting that in habitats with low disturbance, the host might maintain stricter control over its resident microbiota (Sandeu et al., 2022). In contrast, in anthropized areas, we observed an increased richness of genera such as *Exiguobacterium* or *Ideonella*. Evidence suggests that habitat alterations, seasonal changes, or environmental exposures could modify the host microbiota (Fofana et al., 2021; Sandeu et al., 2022). However, this pattern differs from that reported by Muturi et al. (2017), where pesticide use in agricultural landscapes significantly reduces microbial diversity, selectively favoring pesticide degrading taxa, such as *Hydrogenophaga*.

When contrasting bacterial assemblages between species, *An. pseudopunctipennis* presented a more conserved community dominated by *Thorsellia*. Meanwhile, *An. argyritarsis* showed greater openness toward secondary taxa, suggesting that this species is more permissive or possesses a higher rate of horizontal acquisition from the river environment (Gimonneau et al., 2014; Saab et al., 2020; Duque-Granda et al., 2025). Various authors emphasize that differences in microbiota assembly can also be attributed to the intrinsic characteristics of the hosts (gut physiology, immune response, or feeding behavior) (Gimonneau et al., 2014; Galeano-Castañeda et al., 2019; Saab et al., 2020). In general, the bacterial community structure showed exceptionally high levels of alpha diversity, this has been reported when compared to adult stages (Wang et al., 2011; Boissière et al., 2012; Gimonneau et al., 2014); this difference is attributed to the high ecological complexity of the larval niche.

This dichotomy between species-effect and locality-effect was validated through Bray-Curtis dissimilarity analysis and NMDS ordination (stress = 0.013), where geography and host species explained the largest proportion of variance. Both factors are modulators of the microbial structure, reinforcing the hypothesis that species and locality, through their breeding sites, are determinants of taxonomic turnover. Previous studies in the Neotropics and Africa reinforce this assumption in species of anophelines that cohabit in the same environment (Gimonneau et al., 2014; Duque-Granda et al., 2025). It is important to mention that our findings must be interpreted within the methodological scope of the study. Due to the limited number of samples and replicates, inferential statistical testing is restricted and the evaluation of intra-group variability is prevented. Specifically, PERMANOVA results are treated as indicative patterns, as the analysis cannot calculate intra-group variance to discern whether the observed separation is due to biological factors or stochastic noise. Nevertheless, as an exploratory baseline, these results highlight consistent patterns across habitats and species.

The marked dominance of *Thorsellia*, even in distant localities, suggests a stable and broad association during the aquatic stage of these anopheline species. This pattern suggests that larval midgut serves as an environment that favors colonization by this bacterium. However, its ultimate suitability as a biocontrol tool depends on confirming its transstadial persistence and its ability to colonize the adult midgut. Known advantages of endosymbionts, as in the case of *Wolbachia*, lie in their capacity to reduce the prevalence and intensity of *Plasmodium* infection (Gomes et al., 2017). The identification of taxa present in low proportions, also reveals a documented potential for biological control. Among the genera detected with aptitude for paratransgenesis or anti-Plasmodium activity, *Pseudomonas, Aeromonas, Acinetobacter, Chryseobacterium*, and *Bacillus* stand out (Gendrin and Christophides, 2013; Mancini et al., 2018; Liu et al., 2024). Likewise, genera of epidemiological and functional interest were identified, such as *Stenotrophomonas, Hydrogenophaga, Xanthobacter*, and *Azoarcus* (Muturi et al., 2017; Francis et al., 2024; Liu et al., 2024; Duque-Granda et al., 2025). The persistence of these taxa during metamorphosis is significant, as their stability directly influences adult biology and vectorial competence.

This research establishes a baseline of information for future studies that corroborate the stability and persistence of these bacterial taxa across different life stages and climatic seasons of *An. pseudopunctipennis* and *An. argyritarsis*, where the capacity for transstadial transmission has already been evidenced in other anopheline species (Briones et al., 2008; Favia et al., 2008; Wang et al., 2011).

## 5 Conclusion

These findings provide a preliminary basis for understanding the microbiota composition of two medically important species in the country, highlighting the remarkable stability of *Thorsellia* across different ecological settings. Despite the challenges inherent to the sampling scope and to establishing host-symbiont relationships, *Thorsellia* is a genus that warrants further tracking across different developmental stages, along with secondary bacteria that, although detected in lower proportions, have shown effects on the parasite in adult stages. Under the holobiont perspective, understanding how the microbiota influences the fitness, longevity, and resilience of the vector will allow for the orientation of various efforts toward control and surveillance strategies. Following the elimination of indigenous malaria transmission in the country, the vector persists and the risk of re-emergence is latent; therefore, understanding the structure of bacterial communities is essential for developing future alternative strategies for integrated vector management.

## Data Availability

The original contributions presented in the study are publicly available. This data can be found here: NCBI SRA, accession PRJNA1506130.
