# Supplementary material for: Thorsellia dominance and bacterial community structure in two major malaria vectors: an exploratory study in Northwestern Argentina
Source: Front Microbiol. 2026 Sep 23;17:1895126. doi: 10.3389/fmicb.2026.1895126 (PMC13642848; doi:10.3389/fmicb.2026.1895126)
Supplement: Supplementary file 1 [file Data_Sheet_1.docx]

Supplementary Material

# 1 Supplementary Figures and Tables

## 1.1 Supplementary Figures

**Supplementary Figure 1.** Rarefaction curves of bacterial ASV richness across mosquito gut samples

**Supplementary Figure 2.** Relative abundance of key bacterial genera in the larval gut of *Anopheles argyritarsis* (An. arg) and *An. pseudopunctipennis* (An. pse) by river. The heatmap displays the presence and proportion of the five most representative genera identified across three river systems. Red color intensity represents the percentage of relative abundance, ranging from low levels (light shades) to levels exceeding 75% (deep red).

## 1.2 Supplementary Tables

**Supplementary Table 1.** Sequencing data processing and read filtering statistics for Anopheles argyritarsis and Anopheles pseudopunctipennis

| **Sample** | **Raw reads (post_DADA2)** | **After filtering** | **After contaminant removal** | **Rarefied reads** | **Retained reads (%)** |
| --- | --- | --- | --- | --- | --- |
| *Anopheles argyritarsis* |  |  |  |  |  |
| Iruya River | 44503 | 42497 | 42118 | 15991 | 94.6 |
| Conchas River | 27297 | 17602 | 16647 | 15991 | 61 |
| Chirimayo River | 52765 | 16349 | 15991 | 15991 | 30.3 |
|  |  |  |  |  |  |
| *Anopheles pseudopunctipennis* |  |  |  |  |  |
| Iruya River | 46815 | 45873 | 45577 | 15991 | 97.4 |
| Conchas River | 22746 | 18752 | 18457 | 15991 | 81.2 |
| Chirimayo River | 49820 | 43372 | 42871 | 15991 | 86.1 |

**Supplementary Table 2.** Composition and mean relative abundance of bacterial phyla across different *Anopheles* vector species and localities

| **Phylum composition** | | |
| --- | --- | --- |
| **Category** | **Phylum** | **Mean relative abundance (%)** |
| Overall | Proteobacteria | 94.1 |
|  | Bacteroidota | 3.28 |
|  | Firmicutes | 0.89 |
|  | Actinobacteriota | 0.71 |
|  | Planctomycetota | 0.32 |
|  | Cyanobacteria | 0.3 |
|  | Verrucomicrobiota | 0.21 |
|  | Patescibacteria | 0.07 |
|  | Fusobacteriota | 0.06 |
|  | Bdellovibrionota | 0.05 |
|  | WPS-2 | 0.02 |
|  | Myxococcota | 0.01 |
| **By species** |  |  |
| *Anopheles argyritarsis* | Proteobacteria | 91.6 |
|  | Bacteroidota | 4.94 |
|  | Firmicutes | 1.5 |
|  | Actinobacteriota | 0.76 |
|  | Planctomycetota | 0.45 |
|  | Cyanobacteria | 0.35 |
|  | Fusobacteriota | 0.12 |
|  | Verrucomicrobiota | 0.12 |
|  | Bdellovibrionota | 0.1 |
|  | Patescibacteria | 0.05 |
|  | Myxococcota | 0.01 |
|  | WPS-2 | 0.01 |
| *Anopheles pseudopunctipennis* | Proteobacteria | 96.55 |
|  | Bacteroidota | 1.63 |
|  | Actinobacteriota | 0.65 |
|  | Verrucomicrobiota | 0.31 |
|  | Firmicutes | 0.28 |
|  | Cyanobacteria | 0.25 |
|  | Planctomycetota | 0.2 |
|  | Patescibacteria | 0.09 |
|  | WPS-2 | 0.04 |
|  | Bdellovibrionota | 0.01 |
| **By area** |  |  |
| Conchas River | Proteobacteria | 90.85 |
|  | Bacteroidota | 6.88 |
|  | Actinobacteriota | 0.71 |
|  | Verrucomicrobiota | 0.41 |
|  | Cyanobacteria | 0.31 |
|  | Firmicutes | 0.26 |
|  | Planctomycetota | 0.26 |
|  | Fusobacteriota | 0.18 |
|  | Bdellovibrionota | 0.1 |
|  | WPS-2 | 0.03 |
|  | Patescibacteria | 0.02 |
| Iruya River | Proteobacteria | 97.44 |
|  | Actinobacteriota | 0.81 |
|  | Bacteroidota | 0.68 |
|  | Cyanobacteria | 0.35 |
|  | Firmicutes | 0.35 |
|  | Planctomycetota | 0.18 |
|  | Patescibacteria | 0.11 |
|  | Verrucomicrobiota | 0.05 |
|  | Bdellovibrionota | 0.01 |
|  | Myxococcota | 0.01 |
|  | WPS-2 | 0.01 |
| Chirimayo River | Proteobacteria | 93.94 |
|  | Bacteroidota | 2.29 |
|  | Firmicutes | 2.06 |
|  | Actinobacteriota | 0.6 |
|  | Planctomycetota | 0.52 |
|  | Cyanobacteria | 0.23 |
|  | Verrucomicrobiota | 0.17 |
|  | Patescibacteria | 0.09 |
|  | Bdellovibrionota | 0.06 |
|  | WPS-2 | 0.03 |
|  | Fusobacteriota | 0.01 |
